# The mediating role of sleep quality in the association between job stress and quality of life among medical university employees

**DOI:** 10.1038/s41598-026-56623-1

**Published:** 2026-06-07

**Authors:** Zahra Hosseinkhani, Edris Kakemam, Maryam Hadji, Hassan Taherahmadi, Neda Mohammadi

**Affiliations:** 1 Non-Communicable Diseases Research Center, Research institute for Prevention of Non-Communicable Diseases, Qazvin University of Medical Sciences, Qazvin, Iran; 2 Virtanen Institute for Molecular Sciences, University of Eastern Finland, Kuopio, 70150 Finland; 3 Health Units, Faculty of Social Sciences, Tampere University, Tampere, Finland; 4 Clinical Research Development Unit, Qods Hospital, Qazvin University of Medical Sciences, Qazvin, Iran

**Keywords:** Sleep Quality, Effort-reward imbalance, Job Stress, Quality of Life, Health care, Health occupations, Psychology, Psychology

## Abstract

Job stress is a risk factor for poor quality of life), but the mediating role of sleep quality is underexplored, particularly among medical university employees. This study examined the direct and indirect effects of job stress, measured using the effort-reward imbalance (ERI) model, on quality of life, with sleep quality as a hypothesized mediator. A cross-sectional analysis was conducted using data from 1,250 employees of Qazvin University of Medical Sciences in Iran. Participants completed validated instruments, including the effort-reward imbalance (ERI) questionnaire, the Pittsburgh Sleep Quality Index (PSQI), and the World Health Organization Quality of life Instrument (WHOQOL-BREF). Structural equation modeling was employed to test the mediating role of sleep quality in the relationship between job stress and quality of life. Of the study participants, 61.0% exhibited effort-reward imbalance. This group reported poorer sleep quality and lower quality of life across all domains (physical, psychological, social, environmental). Structural equation modeling revealed that a higher ER ratio had a significant negative direct effect on quality of life (B = -1.31, *p* < 0.001) and a significant negative total effect (B = -2.80, *p* < 0.001). The total effect was partially mediated by global sleep quality (indirect effect B = -1.50, *p* < 0.001). Analysis of specific sleep components identified subjective sleep quality, and daytime dysfunction as significant mediating factors. This study demonstrated that job stress, conceptualized as effort-reward imbalance, is associated with the quality of life through both direct and indirect pathways, with sleep quality serving as a significant mediator. These findings highlight the need for integrated workplace interventions that enhance rewards, manage overcommitment, and promote sleep health to protect the well-being of medical university employees.

**Supplementary Information:**

The online version contains supplementary material available at 10.1038/s41598-026-56623-1.

## Introduction

Quality of life (QoL) is a prominent measure of overall well-being in various populations, including healthcare employees^1^. The World Health Organization conceptualizes quality of life as an individual’s perception of their position in life within their cultural and value systems, relative to their goals and concerns^2^. This multidimensional construct is influenced by factors spanning work, personal life, physical and psychological health, and social and environmental contexts^3^. Epidemiological studies have examined the quality of life in health care organizations, identifying various influencing factors such as job satisfaction^4^, stress^5^, sleep disturbance^6^, and sleep quality^7^.

Among these determinants, job stress is a pervasive occupational health issue. It is associated with increased turnover, diminished job satisfaction^8^, reduced quality of working life, and lower perceived organizational support^9^, all of which are associated with poorer quality of life^5^. The effort-reward imbalance (ERI) model provides a robust theoretical framework for analyzing this stress. It emphasizes the principle of reciprocity in the workplace, suggesting that situations characterized by high effort and low reward are particularly stressful and detrimental to health^10^. Efforts encompass both extrinsic demands (e.g. high workload) and intrinsic drive (e.g. overcommitment), while rewards include financial compensation, social esteem, and occupational status control (e.g., job security, career opportunities)^11^. Substantial evidence links high ERI to lower quality of life among healthcare workers.

Substantial evidence links high effort-reward imbalance, indicating an unfavorable effort-reward ratio, to lower quality of life among healthcare workers^10,12^. Concurrently, job stress is a well-established disruptor of sleep^13^. Poor sleep quality is consistently associated with occupational stress^14,15^, and is itself a critical determinant of quality of life in healthcare populations^16^.

While existing research has examined the pairwise associations among job stress, sleep quality, and quality of life^3,10,12,17,18^, critical gaps persist. Before proceeding, we clarify our conceptualization of the three key constructs. Job stress, within the effort-reward imbalance framework, is defined as a perceived disharmony between what employees invest (efforts such as workload and overcommitment) and what they receive in return (rewards). Sleep quality is conceptualized as a multidimensional construct encompassing subjective sleep satisfaction, sleep latency, duration, efficiency, disturbances, medication use, and daytime dysfunction. quality of life follows the WHO definition as an individual’s perceived position in life across physical, psychological, social, and environmental domains.

This study was motivated by two main gaps in the literature. First, no study to date has quantitatively tested whether sleep quality mediates the effort-reward imbalance–quality of life relationship using structural equation modeling (SEM), particularly among university employees. Second, it remains unknown which specific components of sleep quality drive this mediating effect. To address these gaps, we tested two hypotheses H1: sleep quality (global score) significantly mediates the relationship between effort-reward imbalance and quality of life among university employees, such that higher effort-reward imbalance is associated with poorer sleep quality, which in turn is associated with lower quality of life. H2: Specific components of sleep quality differentially mediate the relationship between effort-reward imbalance and quality of life. Therefore, this study employed SEM to investigate the pathways linking job stress and quality of life among employees of a medical university in Iran. It had two primary aims: first, to test whether sleep quality mediates the relationship between job stress (conceptualized via the effort-reward imbalance model) and quality of life; and second, to identify which specific components of sleep quality are central to this mediating effect.

### Participants and procedure

This descriptive-analytical, cross-sectional study utilized data from the Qazvin Employee Health Cohort Study (QEHC), initiated in 2021 to investigate risk factors for non-communicable diseases^19^. The target population consisted of all permanent employees of Qazvin University of Medical Sciences working across its schools, teaching hospitals, clinics, health centers, emergency services, and administrative units. Using a convenience sampling approach, 1,250 employees were enrolled upon visiting the QEHC center. Demographic, occupational, economic, and behavioral data were collected through interviews conducted by trained interviewers. Clinical assessments were performed by medical specialists. All participants provided informed consent, and the study protocol was approved by by the ethics committee of the Deputy of Research and Technology of the Qazvin University of Medical Sciences (IR.QUMS.REC.1404.098). The questionnaires used in this study demonstrated acceptable validity and reliability.

### Measures

Demographic variables included age, sex, marital status, and educational level. Socioeconomic status was assessed via a wealth index, constructed from data on household assets using Principal Component Analysis (PCA) and categorized into three equal groups (tertiles): low, middle, high.

### Sleep quality

Sleep quality was measured using the Pittsburgh Sleep Quality Index (PSQI). This questionnaire includes 19 items that evaluate seven components: sleep duration, sleep latency, subjective sleep quality, sleep efficiency, daytime dysfunction, sleep disturbances, and use of sleep medications. Each component is scored from 0 (no difficulty) to 3 (severe difficulty), and the scores are summed to produce a global ranging between 0 and 21. Poor sleep quality was defined as a PSQI score greater than five^20^. The Persian version of the PSQI has demonstrated good validity and reliability^21^.

### job stress

The effort-reward imbalance questionnaire was used to assess job stress. This 23-item instrument comprises three components: effort (6 items), reward (11 items), and overcommitment (6 items). Responses are recorded on a 4-point Likert scale. An overall effort-reward imbalance is quantified by the effort-reward ratio (ER ratio), calculated as (effort / reward)*k (k is a correction factor to account for the differing number of items). For analysis, this continuous ratio was dichotomized into a binary variable: balanced (ER ratio ≤ 1) and imbalanced (ER ratio > 1)^22^. Throughout the manuscript, figures, and tables, the term “ER ratio” refers to this dichotomized binary variable. Psychometric assessment confirmed the reliability and validity of the Persian version^23^.

### Quality of life

Quality of life was measured using the 26-item WHOQOL-BREF questionnaire, adapted from the original 1998 version, features a reduced number of domains and improved practicality, which have contributed to its widespread acceptance and translations into multiple languages. It assesses four domains: physical health (7 items), psychological health (6 items), social relationships (3 items), and environment (8 items). Two additional items evaluate general quality of life and health. Items are rated on a 5-point Likert scale, with higher domain scores indicating better quality of life^24^. Only the 24 domain-specific items were used in this analysis. Psychometric assessment confirmed the validity and reliability of the Persian version^25^.

### Statistical analysis

Descriptive statistics are presented as mean ± standard deviation (SD) for continuous variables and as frequency (percentage) for categorical variables. Group differences between employees with (ER ratio > 1) and without (ER ratio ≤ 1) effort-reward imbalance were examined using independent t-tests and chi-square tests, as appropriate.

We assessed the direct, indirect, and total effect of effort-reward ratio and quality of life using the Structural Equation Modeling (SEM), with sleep quality includes as a mediator.

The analysis proceeded in two stages. First, we conducted CFA to evaluate the measurement models for each latent construct (effort, reward, overcommitment, sleep quality, and quality of life). Factor loadings, residual variances, and modification indices were examined. The results of factor loadings, model fit indices for CFA models for all constructs were reported in the appendix (**Supplementary Table ****S1****-S6**). Second, we tested three SEMs to examine direct and indirect pathways. In Model I, we investigated the mediating role of sleep quality in the relationship between each effort-reward imbalance component (effort, reward, and overcommitment) and quality of life. Model II examined the direct and indirect effects of the composite ER ratio on quality of life. Model III built upon Model II by disaggregating global sleep quality into its seven constituent components to pinpoint which specific sleep dimensions served as mediators. We used the DWLS estimator and employed bias-corrected bootstrapping with 5,000 resamples to generate 95% confidence intervals for the indirect effects.

Model goodness of fit was assessed using the chi-square to degrees of freedom ratio (χ²/df), Comparative Fit Index (CFI), Goodness of Fit Index (GFI), Root Mean Square Error of Approximation (RMSEA), and Standardized Root Mean Squared Residual (SRMR). Acceptable model fit was defined by the following approximate thresholds: CFI ≥ 0.95, GFI ≥ 0.95, RMSEA ≤ 0.06, and SRMR ≤ 0.08^26^. All analysis were conducted using R statistical software version 4.4.3.

## Results

A total of 1,250 medical university employees participated in the study, of whom 56.6% were male and 43.4% female. Based on the effort-reward ratio (ER ratio), 39.0% of participants were classified into the balanced group (ER ratio ≤ 1), while 61.0% were in the imbalance group (ER ratio > 1) (Table 1). The mean age of participants was 40.57 ± 8.01 years. Those in the imbalance group were significantly younger than those in the balanced group (39.96 ± 8.04 vs. 41.55 ± 7.90 years, *p* = 0.001). The proportion of males was also higher in the imbalance group compared to the balanced group (60.3% vs. 50.8%, *p* = 0.002). There were no significant differences in wealth status or marital status between the groups; however, educational attainment differed significantly (*p* = 0.001), with a lower proportion of post-graduates in the imbalance group (Table 1).

**Table 1:** Demographic and lifestyle characteristics: distribution and comparison by effort-reward ratio (ER ratio) categories.

Variables	Category	Total (*n* = 1,250)	ER ratio		*P*-value*
			Balance (ER ratio < = 1)	Imbalance (ER ratio > 1)	
Age		40.57 ± 8.01	41.55 ± 7.90	39.96 ± 8.04	0.001
Gender	Male	701 (56.58%)	248 (50.82%)	453 (60.32%)	0.002
	Female	538 (43.42%)	240 (49.18%)	298 (39.68%)	
Wealth status	Poor	409 (33.28%)	147 (30.18%)	262 (35.31%)	0.137
	Middle	410 (33.36%)	165 (33.88%)	245 (33.02%)	
	Rich	410 (33.36%)	175 (35.93%)	235 (31.67%)	
Marital status	Single	181 (14.61%)	79 (16.19%)	102 (13.58%)	0.210
	Married	1058 (85.39%)	409 (83.81%)	649 (86.42%)	
Education	Less than High School	87 (7.02%)	28 (5.74%)	59 (7.86%)	0.001
	Diploma	270 (21.79%)	98 (20.08%)	172 (22.9%)	
	Under Graduate	677 (54.64%)	257 (52.66%)	420 (55.93%)	
	Post Graduate	205 (16.55%)	105 (21.52%)	100 (13.32%)	

The results are reported by n (%) for qualitative variable and quantitative for mean ± SD. *Differences were assessed using T-test for quantitative variables and the associations of qualitative variables were evaluated by chi-square (χ²) tests.

As shown in Table 2, significant differences in quality of life and sleep quality were observed between the effort-reward balance and imbalance groups. Participants in the balanced group reported significantly higher mean scores across all quality of life domains: physical health (68.80 ± 16.55 vs. 62.47 ± 17.64, *p* < 0.001), psychological health (71.10 ± 13.37 vs. 67.03 ± 14.88, *p* < 0.001), social relations (69.08 ± 15.01 vs. 66.47 ± 15.61, *p* = 0.003), and environment (62.52 ± 11.26 vs. 56.37 ± 13.13, *p* < 0.001). Conversely, the imbalance group exhibited significantly poorer global sleep quality, as indicated by a higher mean PSQI score (6.79 ± 3.77 vs. 5.59 ± 3.44, *p* < 0.001). A higher prevalence of difficulties was also found across most PSQI components (all *p* < 0.01), including subjective sleep quality, sleep latency, sleep efficiency, sleep disturbance, and daytime dysfunction (Table 2). No significant between-group difference was observed for the use of sleep medication (*p* = 0.892).

**Table 2:** Comparing quality of life and sleep quality by effort-reward ratio.

Variables	components	Levels	Total (*n* = 1,250)	ER ratio		*P*-value*
				Balance (ER ratio < = 1)	Imbalance (ER ratio > 1)	
Quality of life	Physical health		64.98 ± 17.49	68.80 ± 16.55	62.47 ± 17.64	< 0.001
	Psychology health		68.63 ± 14.43	71.10 ± 13.37	67.03 ± 14.88	< 0.001
	Social relations		67.49 ± 15.42	69.08 ± 15.01	66.47 ± 15.61	0.003
	Environment		58.80 ± 12.78	62.52 ± 11.26	56.37 ± 13.13	< 0.001
Total sleep quality			6.30 ± 3.69	5.59 ± 3.44	6.79 ± 3.77	< 0.001
Sleep quality components	Subjective sleep quality	No difficulty	471 (38.2%)	225 (46.2%)	246 (32.98%)	< 0.001
		Mild difficulty	577 (46.8%)	208 (42.71%)	369 (49.46%)	
		Moderate difficulty	128 (10.38%)	41 (8.42%)	87 (11.66%)	
		Severe difficulty	57 (4.62%)	13 (2.67%)	44 (5.9%)	
	Sleep latency	No difficulty	419 (33.98%)	210 (43.12%)	209 (28.02%)	< 0.001
		Mild difficulty	592 (48.01%)	216 (44.35%)	376 (50.4%)	
		Moderate difficulty	107 (8.68%)	30 (6.16%)	77 (10.32%)	
		Severe difficulty	115 (9.33%)	31 (6.37%)	84 (11.26%)	
	Sleep duration	No difficulty	86 (6.97%)	39 (8.01%)	47 (6.3%)	0.005
		Mild difficulty	480 (38.93%)	211 (43.33%)	269 (36.06%)	
		Moderate difficulty	336 (27.25%)	130 (26.69%)	206 (27.61%)	
		Severe difficulty	331 (26.85%)	107 (21.97%)	224 (30.03%)	
	Sleep efficiency	No difficulty	684 (55.52%)	304 (62.42%)	380 (51.01%)	0.002
		Mild difficulty	175 (14.2%)	53 (10.88%)	122 (16.38%)	
		Moderate difficulty	135 (10.96%)	50 (10.27%)	85 (11.41%)	
		Severe difficulty	238 (19.32%)	80 (16.43%)	158 (21.21%)	
	Sleep disturbance	No difficulty	129 (10.46%)	65 (13.35%)	64 (8.58%)	0.003
		Mild difficulty	1011 (82%)	395 (81.11%)	616 (82.57%)	
		Moderate difficulty	92 (7.46%)	26 (5.34%)	66 (8.85%)	
		Severe difficulty	1 (0.08%)	1 (0.21%)	0 (0%)	
	Use of sleep medication	No difficulty	1145 (92.86%)	455 (93.43%)	690 (92.49%)	0.892
		Mild difficulty	39 (3.16%)	13 (2.67%)	26 (3.49%)	
		Moderate difficulty	18 (1.46%)	7 (1.44%)	11 (1.47%)	
		Severe difficulty	31 (2.51%)	12 (2.46%)	19 (2.55%)	
	Daytime dysfunction	No difficulty	491 (39.82%)	232 (47.64%)	259 (34.72%)	< 0.001
		Mild difficulty	552 (44.77%)	193 (39.63%)	359 (48.12%)	
		Moderate difficulty	167 (13.54%)	55 (11.29%)	112 (15.01%)	
		Severe difficulty	23 (1.87%)	7 (1.44%)	16 (2.14%)	

The results are reported by n (%) for qualitative variable and quantitative for mean ± SD. *Differences were assessed using T-test for quantitative variables and the associations of qualitative variables were evaluated by chi-square (χ²) tests.

The CFA results demonstrate acceptable model fit. We have included the full results as Supplementary Tables (**Supplementary Table ****S1****-S5**). Confirmatory factor analysis (CFA) was conducted to assess the measurement properties of each questionnaire. For the quality of life scale (WHOQOL-BREF), the model showed excellent fit: χ²(2) = 2.71, CFI = 0.999, GFI = 1.00, TLI = 0.999, RMSEA = 0.017, SRMR = 0.017. All items loaded significantly on their respective factors (*p* < 0.001), with standardized loadings ranging from 0.662 to 0.877. For the sleep quality scale, model fit was good: χ²(13) = 39.47, CFI = 0.997, GFI = 0.998, TLI = 0.997, RMSEA = 0.041, SRMR = 0.050. Standardized factor loadings ranged from 0.289 to 0.904, all significant (*p* < 0.001), with subjective sleep quality showing the highest loading (0.904). For the Effort scale, model fit was acceptable: χ²(8) = 19.79, CFI = 0.981, GFI = 0.995, TLI = 0.981, RMSEA = 0.034, SRMR = 0.021. Standardized loadings ranged from 0.253 to 0.584, all significant (*p* < 0.001). For the Reward scale, model fit was good: χ²(39) = 209.57; CFI = 0.980; GFI = 0.992; TLI = 0.980; RMSEA = 0.059; SRMR = 0.053. Standardized loadings ranged from 0.240 to 0.826, all significant at *p* < 0.001. For the Overcommitment scale, model fit was good: χ²(9) = 33.31, CFI = 0.981, GFI = 0.991, TLI = 0.981, RMSEA = 0.047, SRMR = 0.024. Standardized loadings ranged from 0.349 to 0.814, all significant (*p* < 0.001).

**Fig. 1:**
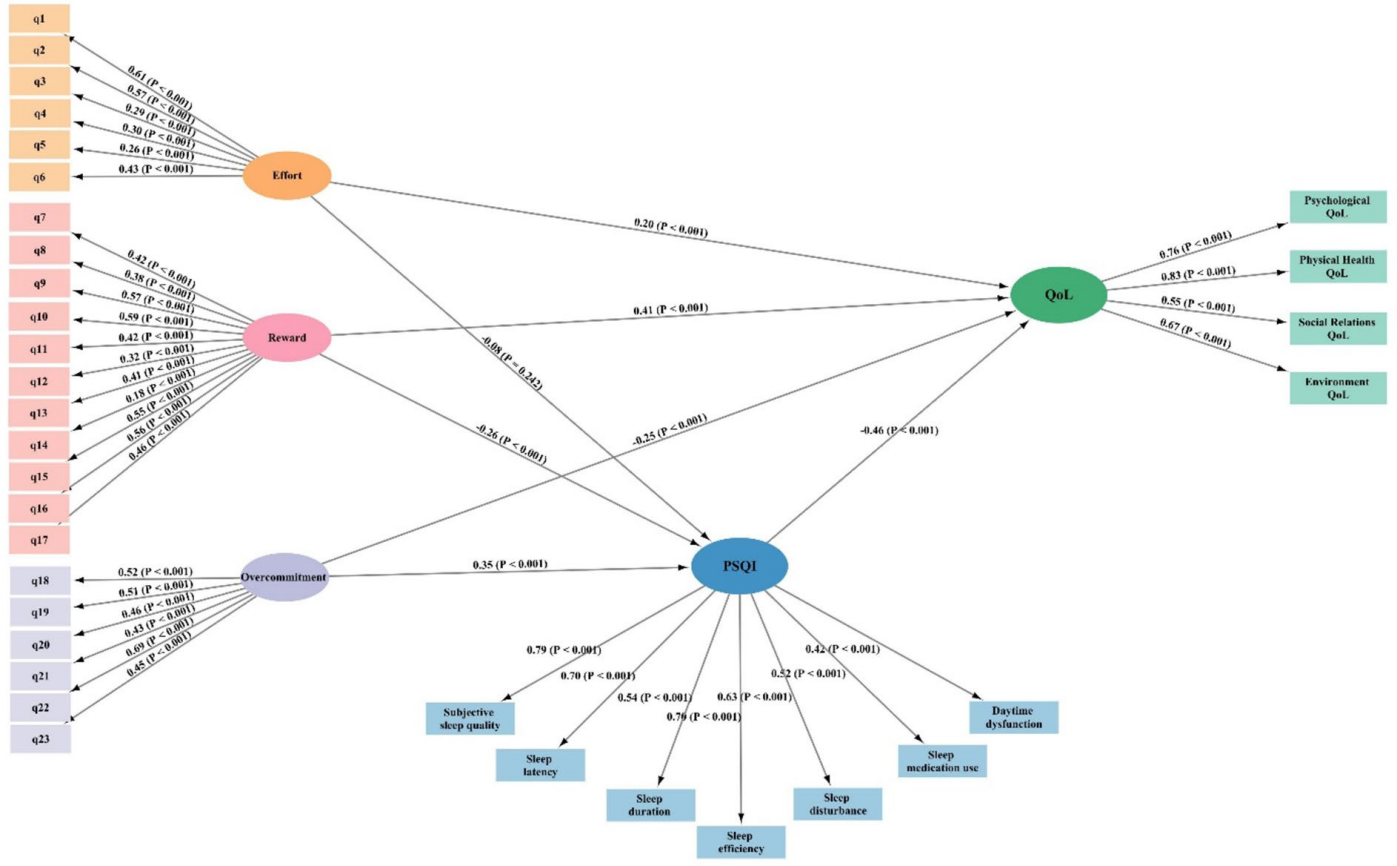
Structural equation model of effort, reward, and overcommitment on quality of life throughout sleep quality (Model I) (Model Fit Indices: χ²(df) = 1324.86 (510); CFI = 0.966; GFI = 0.996; RMSEA = 0.036; SRMR = 0.051).

**Fig. 2:**
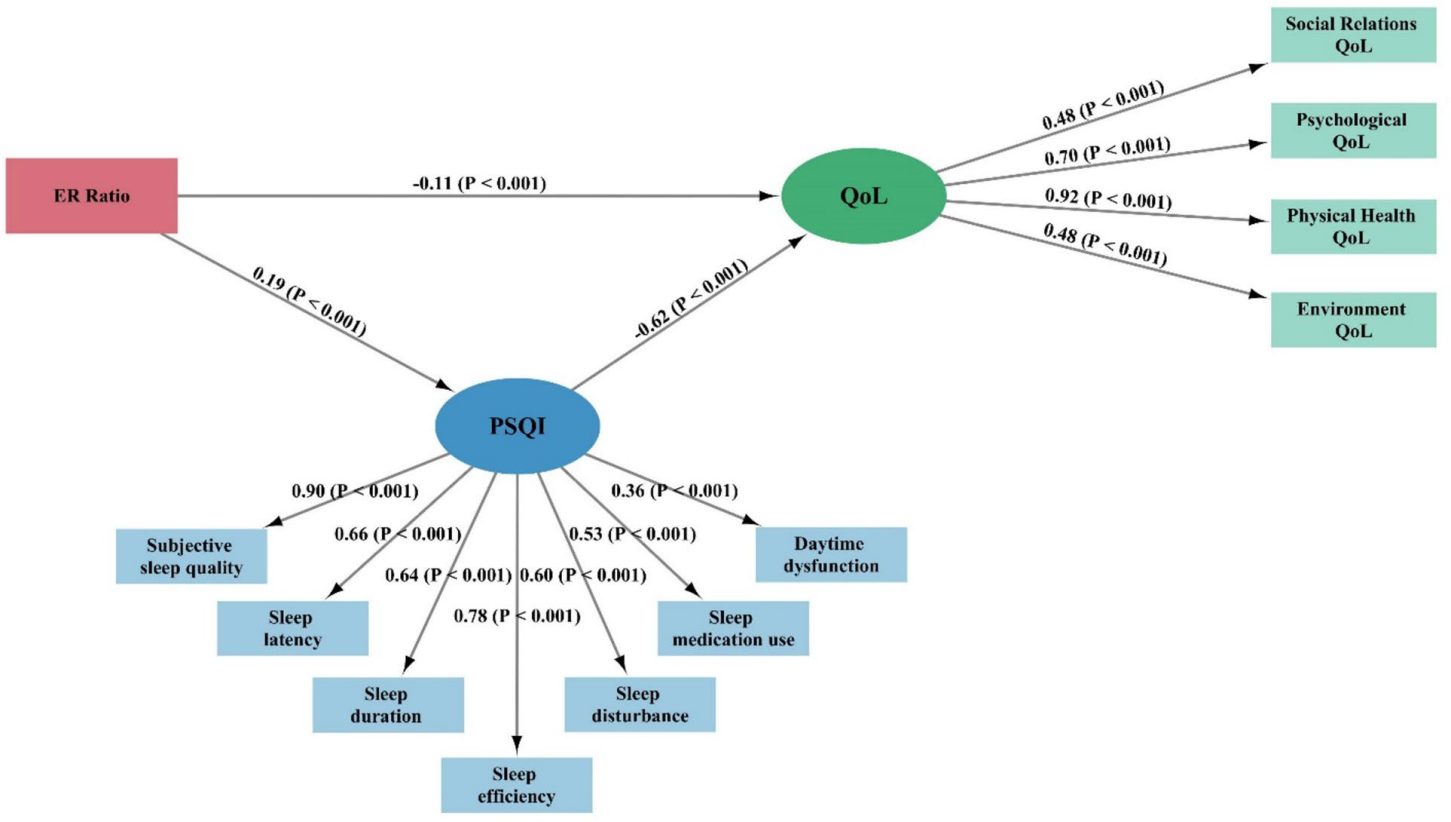
Structural equation model of effort-reward ratio on quality of life throughout sleep quality (Model II) (Model Fit Indices: χ²(df) = 168.60 (48); CFI = 0.992; GFI = 0.994; RMSEA = 0.045; SRMR = 0.048).

**Fig. 3:**
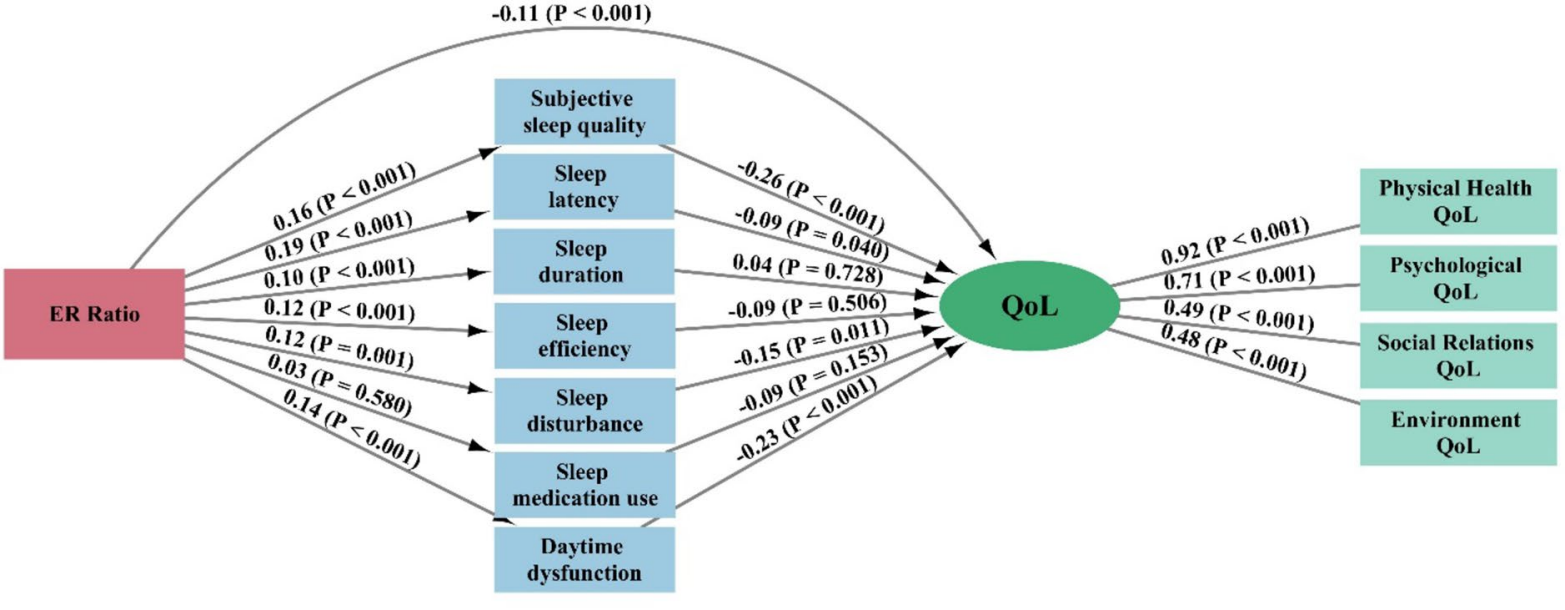
Structural equation model of effort-reward ratio on quality of life throughout components of sleep quality (Model III) (Model Fit Indices: χ²(df) = 36.30 (23); CFI = 0.994; GFI = 0.996; RMSEA = 0.022; SRMR = 0.015).

Three structural equation models were tested to examine the direct and indirect (mediated) effects of job stress on quality of life (Table 3; Figs. 1, 2 and 3). All models demonstrated excellent fit to the data (CFI > 0.95, RMSEA < 0.06, SRMR < 0.08) (Table 3).

**Table 3:** Evaluating direct, indirect, and total effects of models I-III.

Models		Model Pathways	B	95%CI	SB	*P*-value
Model I						
	**Total**					
		Effort → QoL	3.07	(1.79, 5.75)	0.24	0.001
		Reward → QoL	13.42	(10.62, 17.26)	0.54	< 0.001
		Overcommitment → QoL	-7.81	(-13.99, -3.80)	-0.33	< 0.001
	**Direct**					
		Effort → QoL	2.58	(1.20, 4.75)	0.20	0.002
		Reward → QoL	10.37	(7.88, 13.86)	0.41	< 0.001
		Overcommitment → QoL	-4.82	(-7.89, -2.33)	-0.25	< 0.001
	**Indirect**					
		Effort → PSQI → QoL	0.49	(-0.30, 1.27)	0.04	0.232
		Reward → PSQI → QoL	3.05	(2.19, 5.18)	0.12	< 0.001
		Overcommitment → PSQI → QoL	-2.99	(-6.17, -1.43)	-0.09	0.002
Model II						
	**Total**					
		ER ratio → QoL	-2.80	(-4.07, -2.01)	-0.23	< 0.001
	**Direct**					
		ER ratio → QoL	-1.31	(-2.20, -0.45)	-0.11	< 0.001
	**Indirect**					
		ER ratio → PSQI → QoL	-1.50	(-2.18, -0.99)	-0.12	< 0.001
Model III						
	**Total**					
		ER ratio → All sleep components → QoL	-2.79	(-4.07, -2.03)	-0.23	< 0.001
	**Direct**					
		ER ratio → QoL	-1.34	(-2.22, -0.42)	-0.11	< 0.001
	**Indirect**					
		ER ratio → SSQ → QoL	-0.52	(-1.13, -0.20)	-0.04	0.017
		ER ratio → SL → QoL	-0.21	(-0.75, 0.01)	-0.02	0.133
		ER ratio → SDI →QoL	0.05	(-0.23, 0.37)	0.00	0.764
		ER ratio → SDU → QoL	-0.13	(-0.69, 0.21)	-0.01	0.553
		ER ratio → SE → QoL	-0.22	(-0.64, -0.02)	-0.02	0.075
		ER ratio → SM → QoL	-0.03	(-0.37, 0.06)	0.00	0.629
		ER ratio → DD → QoL	-0.39	(-0.68, -0.24)	-0.03	< 0.001

**Abbreviations**: ER ratio=effort-reward ratio; QoL= Quality of Life; SSQ=Subjective sleep quality; SL=Sleep latency; SDI=Sleep disturbance; SDU=Sleep duration; SE= Sleep efficiency; SM=Sleep medication use; DD=Daytime dysfunction; B= Beta Regression Coefficient; CI= Confidence Interval; SB = Standardized Beta Regression Coefficient.

Model I examined the effects of the three effort-reward imbalance components, effort, reward and overcommitment, on quality of life, with sleep quality as a mediator. Reward showed the strongest positive total effect on quality of life (B = 13.42, SB = 0.54, *p* < 0.001), followed by effort (B = 3.07, SB = 0.24, *p* = 0.001). Overcommitment had a significant negative total effect (B = -7.81, SB = -0.33, *p* < 0.001). The direct effects of all three components on quality of life remained significant. Significant indirect effects through sleep quality were found for Reward (B = 3.05, SB = 0.12, *p* < 0.001) and overcommitment (B = -2.99, SB = -0.09, *p* = 0.002), but not for effort (*p* = 0.236). (Fig. 1).

Model II tested the composite ER ratio as the predictor. The ER ratio had a significant negative total effect on quality of life (B = -2.80, SB = -0.23, *p* < 0.001), indicating that employees in the imbalance group (ER ratio > 1) had a quality of life score that was 2.80 units lower than those in the balanced group. This effect was partially mediated by global sleep quality, with a significant direct effect (B = -1.31, SB = -0.11, *p* < 0.001) and a significant indirect effect (B = -1.50, SB = -0.12, *p* < 0.001) (Fig. 2).

Model III extended Model II by decomposing global sleep quality into its seven components to identify specific mediating pathways. The total and direct effects of the ER ratio on quality of life remained significant. Among the sleep components, two demonstrated significant indirect effects: subjective sleep quality (B = -0.52, SB = -0.04, *p* = 0.001) and daytime dysfunction (B = -0.39, SB = -0.03, *p* < 0.001). Sleep latency, sleep efficiency, sleep disturbance, sleep duration, and use of sleep medication did not show significant mediation effects (all *p* > 0.05) (Fig. 3).

## Discussion

This study examined the associations between effort-reward imbalance, sleep quality, and quality of life among medical university employees using structural equation modeling. The key finding is that a majority (61%) of participants experienced effort-reward imbalance, and this condition was associated with significantly poorer sleep quality and lower quality of life across all domains. Crucially, our models demonstrated that the detrimental effect of job stress on quality of life occurs through both direct and indirect pathways, with sleep quality serving as a significant partial mediator. To our knowledge, this is the first study to integrate these constructs within a unified mediation framework in this population.

The decomposition of effort-reward imbalance into its core components (Model I) provided a detailed insight. Model I revealed that reward exerted the strongest total positive effect on quality of life (SB = 0.54). This indicates that improvements in perceived rewards—such as recognition, fair salary structures, and career advancement—can translate into meaningful improvements in employees’ well‑being and overall quality of life. This finding aligns with previous evidence showing that adequate rewards buffer physiological and psychological strain and promote positive health outcomes quality of life^11,27^.

In contrast, overcommitment showed a negative total effect on quality of life (SB = − 0.33). The fact that overcommitment also demonstrated a significant indirect effect on quality of life through sleep quality (SB = − 0.09) suggests that individuals with excessive personal investment at work experience measurable deteriorations in sleep architecture and daytime functioning. This aligns with cognitive‑behavioral theory, which posits that maladaptive work preoccupation interferes with the ability to psychologically detach from work, thereby increasing nighttime hyperarousal and sleep fragmentation^28^.

Both reward and overcommitment exerted significant indirect effects on quality of life via sleep, highlighting sleep as a key pathway through which these specific psychosocial factors ultimately influence overall life quality. The non-significant indirect effect for effort suggests that high workload alone may not degrade quality of life through sleep disruption unless it is coupled with low rewards (imbalance) or an overcommitted.

Model II demonstrated that the ER ratio demonstrated a significant negative total effect on quality of life (SB = − 0.23). Global sleep quality accounted for nearly half of this effect (SB of indirect path = − 0.12), representing a meaningful proportion of the mechanism through which job stress translates into poorer quality of life. This confirms and extends prior research that has independently linked high effort-reward imbalance to poor sleep^17^ and reduced quality of life^11^. Our model quantifies the mediation, suggesting that a substantial portion of stress’s impact on well-being is explained by its sleep-disrupting effects.

A deeper analysis using Model III revealed that not all sleep components contributed equally to the mediation. Specifically, subjective sleep quality and daytime dysfunction were identified as the primary mediators. Subjective sleep experience and daytime impairment are clinically relevant indicators strongly associated with stress-related hyperarousal, circadian disruption, and performance deficits^16,29,30^. The fact that sleep latency, sleep efficiency, sleep duration, and sleep disturbance did not contribute significant indirect effects suggests that effort-reward imbalance affects quality of life primarily through workers’ perceptions of sleep restfulness and their ability to function during the day rather than through objective sleep parameters.

The observed relationships are biologically plausible. Chronic exposure to effort-reward imbalance, a potent psychosocial stressor, can lead to sustained activation of the hypothalamic-pituitary-adrenal (HPA) axis and elevated cortisol levels^31,32^. This neuroendocrine dysregulation is known to impair sleep architecture, disrupt circadian rhythms^33,34^, and contribute to the subjective experience of non-restorative sleep. Over time, such sleep disturbances can trigger systemic inflammation and allostatic load^35^ decrease quality of life across physical, psychological, and social domains^36^.

Our findings suggest two targeted intervention strategies for medical university employees. First, organizational efforts should prioritize enhancing rewards—such as recognition, fair compensation, and career development—to directly improve quality of life and correct effort-reward imbalance. Second, given the significant mediating role of sleep quality, integrating sleep health programs (e.g., sleep hygiene education, cognitive-behavioral strategies for insomnia) into workplace wellness initiatives offers a direct route to buffer the negative association of job stress and well-being. Addressing both the source of job stress and poor sleep quality represents a synergistic approach to protecting this vital workforce’s quality of life.

Although the results of this study address a gap in the literature, in our study there are several limitations that should be taken into consideration in future research. First, while we used validated questionnaires, all measures relied on self-reports data. Second, focusing exclusively on university medical staff may limit generalizability to other occupational groups with different stress profiles. Third, our study focused exclusively on sleep quality as a mediator. Future research should examine whether active behavioral responses to job stress (e.g., physical activity, substance use, social engagement, …) operate in parallel with or interact with sleep quality in influencing quality of life. Fourth, unmeasured confounding factors such as individual chronotype and comorbid health conditions may have influenced the results. Finally, due to the cross-sectional design, the terms ‘effect,’ ‘direct effect,’ and ‘indirect effect’ are used here to reflect the directional assumptions of our structural equation model based on theoretical precedence. Causality cannot be inferred. Future longitudinal studies incorporating objective measures and broader covariate assessment are needed to establish temporal precedence and causal direction.

## Conclusion

This study demonstrates through structural equation modeling that effort-reward imbalance significantly is associated with quality of life among medical university employees both directly and indirectly through sleep quality, with subjective sleep quality, and daytime dysfunction emerging as key mediating factors. Furthermore, analysis of the effort-reward imbalance components revealed that reward showed the most substantial protective association with quality of life, while overcommitment displayed the strongest detrimental relationship with sleep quality. These results underscore the necessity for integrated workplace interventions that simultaneously target the reduction of effort-reward imbalance and promote sleep quality to protect the well-being of university employees.

## Supplementary Information

Below is the link to the electronic supplementary material.

Supplementary Material 1

## Data Availability

The datasets used during the current study are available from the corresponding author on request.

## List of Abbrivations

QoL: Quality of life
ERI: Effort-reward imbalance
PCA: Principal component analysis
PSQI: Pittsburgh Sleep Quality Index
SD: Standard deviation
SEM: Structural equation modeling
CFI: Comparative Fit Index
GFI: Goodness of Fit Index
RMSEA: Root Mean Square Error of Approximation
SRMR: Standardized Root Mean Squared Residual
