# Supplementary material for: The mediating role of sleep quality in the association between job stress and quality of life among medical university employees
Source: Sci Rep. 2026 Jun 7;16:25917. doi: 10.1038/s41598-026-56623-1 (PMC13486691; doi:10.1038/s41598-026-56623-1)
Supplement: Supplementary file 1 [file 41598_2026_56623_MOESM1_ESM.docx]

| **Supplementary Table S1: Confirmatory Factor Analysis of Quality of Life (WHOQOL-BREF)** | | | | | |
| --- | --- | --- | --- | --- | --- |
| Indicator | **Loading (95%CI)** | **Std_Loading** | **Wald** | **P-value** | |
| Environment | 1.000 (1.000, 1.000) | 0.662 | - | - | |
| Social relations | 1.237 (1.083, 1.392) | 0.678 | 15.694 | <0.001 | |
| Physical health | 1.445 (1.278, 1.612) | 0.698 | 16.93 | <0.001 | |
| Psychology health | 1.497 (1.338, 1.656) | 0.877 | 18.453 | <0.001 | |
| **Model Fit Indices:** χ²(df) = 2.71(2); CFI = 0.999; GFI = 1.00; TLI = 0.999; RMSEA = 0.017; SRMR = 0.017 | | | | |  |

| **Supplementary Table S2: Confirmatory Factor Analysis of Sleep Quality (PSQI)** | | | | |
| --- | --- | --- | --- | --- |
| Indicator | **Loading (95%CI)** | **Std_Loading** | **Wald** | **P-value** |
| Subjective sleep quality | 1.000 (1.000, 1.000) | 0.904 | - | - |
| Daytime dysfunction | 0.319 (0.244, 0.394) | 0.289 | 8.379 | <0.001 |
| Sleep disturbance | 0.611 (0.540, 0.682) | 0.553 | 16.938 | <0.001 |
| Sleep duration | 0.732 (0.670, 0.794) | 0.662 | 23.194 | <0.001 |
| Sleep efficiency | 0.893 (0.832, 0.954) | 0.808 | 28.799 | <0.001 |
| Sleep latency | 0.729 (0.671, 0.787) | 0.659 | 24.643 | <0.001 |
| Use of sleep medication | 0.561 (0.455, 0.667) | 0.507 | 10.327 | <0.001 |
| **Model Fit Indices:** χ²(df) = 39.47(13); CFI = 0.997; GFI = 0.998; TLI = 0.997; RMSEA = 0.041; SRMR = 0.050 | | | | |

| **Supplementary Table S3: Confirmatory Factor Analysis of Effort (ERI Questionnaire)** | | | | |
| --- | --- | --- | --- | --- |
| **Indicator** | **Loading (95%CI)** | **Std_Loading** | **Wald** | **P-value** |
| ER1 | 1.000 (1.000, 1.000) | 0.559 | - | - |
| ER2 | 0.559 (0.447, 0.672) | 0.355 | 9.745 | <0.001 |
| ER3 | 0.808 (0.647, 0.969) | 0.521 | 9.828 | <0.001 |
| ER4 | 0.741 (0.587, 0.895) | 0.400 | 9.444 | <0.001 |
| ER5 | 0.476 (0.323, 0.629) | 0.253 | 6.096 | <0.001 |
| ER6 | 1.057 (0.861, 1.253) | 0.584 | 10.55 | <0.001 |
| **Model Fit Indices:** χ²(df) = 19.79 (8); CFI = 0.981; GFI = 0.995; TLI = 0.981; RMSEA = 0.034; SRMR = 0.021 | | | | |

| **Supplementary Table S4: Confirmatory Factor Analysis of Reward (ERI Questionnaire)** | | | | |
| --- | --- | --- | --- | --- |
| **Indicator** | **Loading (95%CI)** | **Std_Loading** | **Wald** | **P-value** |
| ER7 | 1.000 (1.000, 1.000) | 0.644 | NA | NA |
| ER8 | 0.698 (0.633, 0.762) | 0.450 | 21.234 | <0.001 |
| ER9 | 1.282 (1.188, 1.377) | 0.826 | 26.615 | <0.001 |
| ER10 | 1.163 (1.088, 1.238) | 0.749 | 30.447 | <0.001 |
| ER11 | 0.688 (0.628, 0.748) | 0.443 | 22.591 | <0.001 |
| ER12 | 0.483 (0.425, 0.540) | 0.311 | 16.373 | <0.001 |
| ER13 | 0.638 (0.577, 0.699) | 0.411 | 20.627 | <0.001 |
| ER14 | 0.373 (0.319, 0.428) | 0.240 | 13.445 | <0.001 |
| ER15 | 1.236 (1.141, 1.332) | 0.797 | 25.405 | <0.001 |
| ER16 | 0.982 (0.914, 1.051) | 0.633 | 28.062 | <0.001 |
| ER17 | 0.591 (0.525, 0.658) | 0.381 | 17.452 | <0.001 |
| **Model Fit Indices of reward:** χ²(df) = 209.57 (39); CFI = 0.980; GFI = 0.992; TLI = 0.980; RMSEA = 0.059; SRMR = 0.053 | | | | |
|  |  |  |  |  |
|  |  |  |  |  |

| **Supplementary Table S5: Confirmatory Factor Analysis of Overcommitment (ERI Questionnaire)** | | | | |
| --- | --- | --- | --- | --- |
| **Indicator** | **Loading (95%CI)** | **Std_Loading** | **Wald** | **P-value** |
| ER18 | 1.000 (1.000, 1.000) | 0.349 | - | - |
| ER19 | 2.231 (1.785, 2.677) | 0.619 | 9.81 | <0.001 |
| ER20 | 1.276 (0.952, 1.600) | 0.429 | 7.724 | <0.001 |
| ER21 | 1.738 (1.357, 2.119) | 0.477 | 8.939 | <0.001 |
| ER22 | 2.837 (2.258, 3.415) | 0.814 | 9.611 | <0.001 |
| ER23 | 2.089 (1.646, 2.532) | 0.584 | 9.239 | <0.001 |
| **Model Fit Indices of overcommitment:** χ²(df) = 33.31 (9); CFI = 0.981; GFI = 0.991; TLI = 0.981; RMSEA = 0.047; SRMR = 0.024 | | | | |
|  |  |  |  |  |
